# *Staphylococcus argenteus*: An emerging subclinical bovine mastitis pathogen in Thailand

**DOI:** 10.14202/vetworld.2019.1940-1944

**Published:** 2019-12-12

**Authors:** Natapol Pumipuntu

**Affiliations:** One Health Research Unit, Faculty of Veterinary Sciences, Mahasarakham University, Maha Sarakham, Thailand

**Keywords:** mass spectrometry, *Staphylococcus argenteus*, *Staphylococcus aureus* complex, subclinical bovine mastitis

## Abstract

**Background and Aim:**

*Staphylococcus argenteus* is an emerging species of the *Staphylococcus aureus* complex. It has usually been misidentified as *S. aureus* by conventional methods and its characteristics. *S. argenteus* is potentially emerging in both humans and animals with an increasing global distribution. This study aimed to differentiate and identify *S. argenteus* from *S. aureus* collected and isolated from milk samples of subclinical bovine mastitis cases in Maha Sarakham Province, Northeastern Thailand.

**Materials and Methods:**

Forty-two isolates of *S. aureus* were studied from 132 individual milk samples collected from subclinical bovine mastitis cases of 15 dairy farms in three districts of Maha Sarakham, Thailand. The identification was confirmed by conventional and immune-agglutination methods. Fifteen representative isolates which were suspected as being *S. argenteus* were analyzed by matrix-assisted laser desorption/ionization time-of-flight mass spectrometry (MALDI-TOF MS).

**Results:**

The result from MALDI-TOF MS confirmed that seven from 15 isolates were *S. argenteus* and eight isolates were *S. aureus*.

**Conclusion:**

This study indicated that MALDI-TOF MS used as an identification and classification method could accurately differentiate the novel species, *S. argenteus*, from the *S. aureus* complex which is usually misdiagnosed. In addition, the identification of *S. argenteus* seems to be very limited in technical difficulty despite the fact that it may be the important causative pathogen in bovine mastitis as well as a pathogenic bacterium in food and milk. Therefore, it is essential for both bovine medicine and veterinary public health to emphasize and recognize this bacterial pathogen as an emerging disease of staphylococcal bacteria that there is a need for further study of *S. argenteus* infections.

## Introduction

Bovine mastitis is the most prevalent and costly disease in dairy cows and the livestock milk industry. Losses or reductions in dairy cow milk production arise from factors such as changes in milk composition, discarded or low-quality milk and culling, increases in veterinary services, and increases in labor costs [1]. Bovine mastitis can be classified as clinical mastitis, wherein inflammation appears on the udders of dairy cows, and subclinical mastitis, wherein there are no visible signs of inflammation or infection. The disease is mainly caused by *Staphylococcus aureus*, which is responsible not only for contagious mastitis but also for environmental mastitis [2]. Bovine mastitis caused by staphylococcal infections especially in *S. aureus* infections have become a crucial concern in the dairy farm industry worldwide, including Thailand. It is essential that we understand the prevalence and epidemiology of *Staphylococcus* species to develop programs and strategies for controlling and preventing this infectious disease in dairy farming areas worldwide [2].

Many incidences of mastitis are directly detected based on high somatic cell counts (SCCs) in individual cows through data recording of farms and the California mastitis test (CMT) [3]. The majority of the somatic cells are leukocytes that are observed in milk in increasing numbers, usually as an immune response by the animal to a mastitis-causing pathogen. SCCs can be used to diagnose subclinical mastitis cases in herds that display no obvious clinical symptoms of the illness and no visible changes in the composition of milk [3]. Subclinical mastitis is 40 times more common than clinical mastitis [4]; its importance lies mainly in that it is often a hidden or invisible problem for the herd, requiring more concern and more vigilance than clinical mastitis.

Recently, the use of many new methods for bacterial identification helped to differentiate strains or groups of strains and to elucidate the genetic structure of *S. aureus*. These methods include whole-genome sequencing, staphylococcal protein A typing, staphylococcal cassette chromosome *mec* typing, multilocus sequence typing (MLST), core genome MLST, and matrix-assisted laser desorption/ionization time-of-flight mass spectrometry (MALDI-TOF MS) [5-10]. MALDI-TOF MS is a technology that can be applied to identify bacteria using the distinction of their protein peaks [11], the differentiation of spectra or signatures of cell extracts [12], and whole cells [13]. Therefore, it has been purposed in many studies as a tool to differentiate *Staphylococcus argenteus* from *S. aureus*. This research revealed the novel staphylococcal species *S. argenteus*, which belongs to clonal complex 75 and related ST [14]. Its phenotype is similar to that of *S. aureus*, i.e., it is Gram-positive cocci in clusters, catalase-positive, and coagulase-positive and shows β-hemolysis on blood agar. In fact, *S. argenteus* likely has been misidentified as *S. aureus*. Nonetheless, it has a dominant characteristic that is different from the characteristics of *S. aureus*, i.e., it has white-colored colonies or a non-pigmented appearance on blood agar due to the lack of staphyloxanthin, which is a carotenoid pigment providing the yellowish or golden color observed in *S. aureus* colonies [14]. At present, *S. argenteus* has been clinically isolated from humans and is widespread, i.e. there are many reports of its prevalence in patients from many countries [15]. However, reports of *S. argenteus* that have been isolated from animals are limited, particularly in comparison to a large number of reports of the species isolated from livestock. This bacterium has not been detected in bovine mastitis cases until now; however, previous reports have assumed that *S. argenteus* was the cause of some diseases that have been wrongly identified to have been caused by *S. aureus* [16].

This study aimed to investigate the prevalence of *S. aureus* and *S. argenteus* infections in milk samples obtained from bovines affected by subclinical mastitis on dairy farms in Maha Sarakham, Northeast Thailand. The molecular diversity of bacterial isolates was determined using MALDI-TOF MS and MLST methods to establish the prevalence and accurate typing for *S. aureus* and *S. argenteus*. This molecular epidemiology data will be invaluable for developing appropriate alternative control and prevention strategies for these pathogens, particularly with respect to the emergence of a new pathogen in dairy herds for which there is currently very limited data in Thailand.

## Materials and Methods

### Ethical approval

All research procedures performed in this study were approved by the Faculty of Tropical Medicine-Animal Care and Use Committee (FTM-ACUC), Mahidol University, Thailand (FTM-ACUC 005/2016).

### Sample collection and preparation

A total of 132 individual milk samples were collected from subclinical mastitis-affected cattle using aseptic techniques. Each individual milk sample was collected from four quarters (bulk milk) of a cow. The classification of milk samples was confirmed by positive CMT and SCC screenings. Samples were collected from 15 dairy farms from Muang, Kantarawichai, and Borabue district in Maha Sarakham Province, Northeast Thailand. All samples were collected between September 2017 and April 2018. All milk samples were subjected to bacterial culturing on Baird-Parker Agar supplemented with egg yolk Tellurite Emulsion (Oxoid, Basingstoke, UK), a selective media for coagulase-positive staphylococci. Cultures were incubated at 37°C for 24 h. Suspected bacterial colonies were identified as *S. aureus* using various conventional methods [17] such as Gram staining, the catalase enzyme test, Mannitol Salt Agar (MSA) test, tube coagulase test (TCT), the deoxyribonuclease (DNase) test, and the agglutination test, which was determined using Dryspot Staphytect Plus Kit (Oxoid, Basingstoke, UK) to confirm *S. aureus*.

### MALDI-TOF MS

Fifteen bacterial isolates comprising ten white colony color isolates and five golden colony color isolates were chosen as representative clinical isolates of each study area to examine the spectra analysis using the spectrometer MALDI-TOF MS Biotyper 3.0 database Ultraflex platform (Bruker Daltonics, Bremen, Germany). For the criteria to select *S. aureus* isolates as representative isolates, all the isolates which had white colony color (10 isolates) were selected first. Then, five isolates of bacterial colonies that had golden color were chosen randomly. However, the criteria for selected five golden colonies were chosen as representative isolates from all three study areas by random in different farms. For sample preparation of each isolate, one colony of bacteria was suspended in 500 ml of 70% ethanol; then, the suspension was centrifuged at 13,000 rpm for 5 min. The supernatant was discarded; the pellet was air-dried and resuspended by adding 25 ml of 70% formic acid solution. The bacterial suspension was gently mixed, and then 25 ml pure acetonitrile was added to the suspension. It was centrifuged for 2 min at 13,000 rpm to extract protein from the pellet [10]. One milliliter of crude protein extract was transferred onto a 96-spot polished steel target plate (Bruker Daltonics), and it was dried at room temperature. The samples were covered with 1 ml α-cyano-4-hydroxycinnamic acid matrix solution (Bruker Daltonics). For positive control and calibration reference, the protocol used 1 ml bacterial test standard (BTS; Bruker Daltonics). The mass range analysis of spectra was performed using the MicroFlex tool (Bruker Daltonics). The analysis was performed in positive linear mode at the range 2000-20,000 m/z. Each spectrum obtained after 300 shots were automatically attained according to the system procedure. The identification of microorganisms was performed in duplicate by analyzing the score that related to the degree of similarity with the reference spectrum contained in the database. According to the score analysis, MALDI Biotyper score range 2.000-2.299 is suggested for secure genus identification or probable species identification, and MALDI Biotyper score 2.300-3.000 is recommended for highly probable species identification. The main spectrum in this protocol was obtained using MALDI Biotyper automated FlexControl software version 3.0 (Bruker Daltonics, https://bruker-daltonics-flexcontrol.software.informer.com/3.0/).

### Statistical analysis

Descriptive statistics in Microsoft Excel version 2013 (IBM, NY, USA) were used to define the occurrence of *S. aureus* and *S. argenteus*.

## Results

### Prevalence of *S. aureus*

Of the 132 milk samples obtained from individual bovine cases affected by mastitis, 28 cases (21.21%) presented with *S. aureus* infection, and 42 *S. aureus* isolates were identified using Gram staining, the catalase enzyme test, MSA test, TCT, the DNase test, and the Dryspot Staphytect Plus Kit (Oxoid, Basingstoke, UK), as shown in Table-1.

**Table-1:** Number of cases and *Staphylococcus aureus* infection cases from subclinical bovine mastitis.

Study area	Subclinical mastitis cases	*Staphylococcus aureus* infection cases	Percentage of occurrence
Maha Sarakham			
Borabue	45	8	17.78
Kantarawichai	72	16	22.22
Muang	15	4	26.67
Total	132	28	21.21

### Identification of *S. argenteus* by MALDI-TOF MS

**Figure-1:**
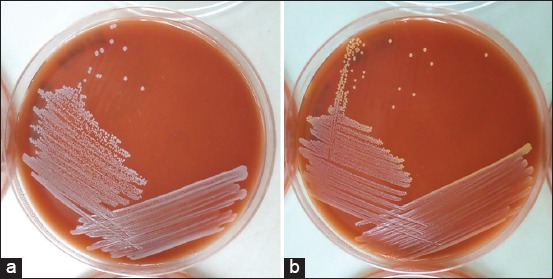
Morphology of staphylococci colony isolates on Columbia agar (Basingstoke, UK). (a) White colony color isolates suspected as *Staphylococcus argenteus*. (b) Golden colony color isolates suspected as *Staphylococcus aureus*.

From 42 *S. aureus* isolates, this study chose 15 representative isolates, including ten white colony color isolates, which were suspected to be *S. argenteus* and five golden colony color isolates on Columbia agar (Oxoid, Basingstoke, UK), as shown in Figure-1 to analyze using MALDI-TOF MS. After visual inspection and receiving the spectra result of their ionizable cell surface components which were compared to the data of spectra in the database from Bruker with in-house databases, the result showed that they were eight isolates which included three white colony color isolates and all five golden colony color isolates that matched to *S. aureus* database. Interestingly, there were seven isolates (all isolates were white colony color) that matched to *S. argenteus* database with high score value (>2.3) and identified by MALDI-TOF MS to be a novel species of staphylococci called *S. argenteus*, as shown in Table-2.

**Table-2:** MALDI-TOF MS result for *Staphylococcus argenteus* identification.

Isolate no.	Colony color	Area	Conventional identification	MALDI-TOF MS identification	Score value (best match)	Score value (2^nd^ best match)
DS060	White	KW	*S. aureus*	*S. aureus*	2.441	2.426
DS039	White	KW	*S. aureus*	*S. argenteus*	2.496	2.12
DS015	Golden	KW	*S. aureus*	*S. aureus*	2.447	2.415
DS021	White	KW	*S. aureus*	*S. argenteus*	2.743	2.238
DS078	Golden	KW	*S. aureus*	*S. aureus*	2.514	2.46
DS066	Golden	KW	*S. aureus*	*S. aureus*	2.558	2.492
SS018	White	BR	*S. aureus*	*S. argenteus*	2.481	2.311
DS005	Golden	BR	*S. aureus*	*S. aureus*	2.46	2.446
DS004	White	BR	*S. aureus*	*S. argenteus*	2.354	2.314
DS003	White	BR	*S. aureus*	*S. argenteus*	2.761	2.301
B001	White	M	*S. aureus*	*S. aureus*	2.503	2.463
B002	Golden	M	*S. aureus*	*S. aureus*	2.209	2.133
S001	White	BR	*S. aureus*	*S. aureus*	2.17	2.115
B021	White	M	*S. aureus*	*S. argenteus*	2.436	2.245
B024	White	M	*S. aureus*	*S. argenteus*	2.431	2.351

*KW=Kantarawichai, BR=Borabue, M=Muang, *S. argenteus*=*Staphylococcus argenteus*, *S. aureus*=*Staphylococcus aureus*, MALDI-TOF MS=Matrix-assisted laser desorption/ionization time-of-flight mass spectrometry

## Discussion

Staphylococcal bacteria are important pathogens that cause bovine mastitis in dairy cows, a subclinical intra-mammary infection resulting in economic losses to the dairy industry worldwide. In addition, milk from infected dairy cows is a major source of enterotoxigenic *S. aureus*, leading to food poisoning and gastrointestinal disease [18]. In Thailand, especially in Maha Sarakham which has intensive dairy farming, *Staphylococcus* spp., particularly *S. aureus*, is still one of the important contagious etiological bacterial agents associated with subclinical infections in dairy cows [19]. This study investigated the prevalence of *S. aureus* infecting the intra-mammary glands of dairy cows in 15 farms from three sub-district areas in Maha Sarakham Province of Thailand. Among 132 subclinical mastitis cases, this study identified 42 isolates from 28 cases as *S. aureus*. It found the occurrence of *S. aureus* infection from subclinical mastitis to be moderately high at 21.21%. This may imply that *S. aureus* has continued to be an important bacterial infection in dairy cow population, causing high rates of subclinical bovine mastitis throughout this study area. This data indicate that *S. aureus* seems to be the common contagious bacterial pathogen of subclinical bovine mastitis in Maha Sarakham. Moreover, Witaya [19] found 8% of *S. aureus* isolates causing bovine mastitis in Chiang Mai. This comparing data showed the high percentage of *S. aureus* infection in bovine mastitis from the past which needs to be concerned for both livestock and veterinary public health. An increase in the prevalence of *S. aureus* infections associated with bovine mastitis has also been reported in other countries; Northwest Iran [20], Jiangsu Province, China [2], Sweden [21], Eastern Algeria [22], and Dharwad, and India [23].

Moreover, the identification of *S. aureus* isolated from subclinical bovine mastitis can be studied using MALDI-TOF MS. This technique has the potential to provide improving more accurate technique for diagnosing bacterial infection and microbial identification. This information relates to a previous study that mentioned that MALDI-TOF MS can support clinicians or veterinarians in making a precise diagnosis of infectious diseases and provide targeted prescriptions, which can reduce the potential risks of antimicrobial resistance [24].

Interestingly, this study found the occurrence of the novel strain of coagulase-positive staphylococci called “*S. argenteus*” that has been isolated from humans in Australia, Cambodia, and Thailand [15,16,25]. Seven isolates from ten isolates (70%) of *S. aureus* which had white colony color were identified to be *S. argenteus* by MALDI-TOF MS. It may provide the first report of *S. argenteus* isolated from bovine mastitis or livestock animal origin in Thailand using MALDI-TOF MS. Therefore, there is a clinical need for further study of *S. aureus* and *S. argenteus* causing bovine mastitis as well as other staphylococcal infections in other animals in case of increased virulence. A comparison between *S. aureus* and *S. argenteus* is needed to define its importance in the veterinary field and for the benefit of public health.

## Conclusion

These findings revealed that the staphylococcal bacterium, *S. aureus*, is still an important pathogen causing subclinical bovine mastitis in three areas of Maha Sarakham, Thailand, with a high prevalence indicating a need for concern over livestock and veterinary public health. The molecular identification of *S. aureus* by MALDI-TOF MS showed higher discriminatory power than conventional techniques. The technique can differentiate *S. aureus* from the other staphylococci. Furthermore, the discovery of *S. argenteus*, a novel strain of coagulase-positive staphylococci, in bovine mastitis seems to be the first report *of S. argenteus* isolated from bovine mastitis milk samples in Thailand using MALDI-TOF MS.

The phenotype of *S. argenteus* is similar to that of *S. aureus*, except for the white colony color. To differentiate *S. argenteus* from *S. aureus*, the molecular-based method MALDI-TOF MS was the most appropriate technique available; therefore, the identification of *S. argenteus* by conventional techniques may lead to misdiagnosing and a false report of *S. aureus*. Thus, the occurrence of the *S. argenteus* isolates from animals, including livestock origin, is likely to have been reported nowhere else. Based on this study, an emerging staphylococcal strain, *S. argenteus*, needs to be investigated regarding its infectious abilities, virulence factors, and antimicrobial susceptibilities. Although this study has documented its association with bovine mastitis, this pathogen may also lead to other types of infections in other animals.

## Author’s Contributions

NP planned and designed the study, collected the samples, did laboratory works and data analysis. Finally, NP drafted, revised, and approved the final manuscript.

## References

[1] Hillerton J.E, Berry E.A (2005). Treating mastitis in the cow a tradition or an archaism. J. Appl. Microbiol.

[2] Xu J, Tan X, Zhang X, Xia X, Sun H (2015). The diversities of staphylococcal species, virulence and antibiotic resistance genes in the subclinical mastitis milk from a single Chinese cow herd. Microb. Pathog.

[3] Schwarz D, Diesterbeck U.S, Failing K, König S, Brügemann K, Zschöck M, Wolter W, Czerny C.P (2010). Somatic cell counts and bacteriological status in quarter foremilk samples of cows in Hesse, Germany a longitudinal study. J. Dairy Sci.

[4] Islam M.A, Islam M.Z, Islam M.A, Rahman M.S, Islam M.T (2011). Prevalence of subclinical mastitis in dairy cows in selected areas of Bangladesh. Bangladesh J. Vet. Med.

[5] Park K.H, Greenwood-Quaintance K.E, Uhl J.R, Cunningham S.A, Chia N, Jeraldo P.R, Sampathkumar P, Nelson H, Patel R (2017). Molecular epidemiology of *Staphylococcus aureus* bacteremia in a single large Minnesota medical center in 2015 as assessed using MLST, core genome MLST and spa typing. PLoS One.

[6] Manara S, Pasolli E, Dolce D, Ravenni N, Campana S, Armanini F, Asnicar F, Mengoni A, Galli L, Montagnani C, Venturini E, Rota-Stabelli O, Grandi G, Taccetti G, Segata N (2018). Whole-genome epidemiology, characterisation, and phylogenetic reconstruction of *Staphylococcus aureus* strains in a paediatric hospital. Genome Med.

[7] Shrivastava N, Sharma V, Shrivastav A, Nayak A, Rai A.K (2018). Prevalence and characterization of panton-valentine leukocidin-positive *Staphylococcus aureus* in bovine milk in Jabalpur district of Madhya Pradesh, India. Vet. World.

[8] Vishnupriya S, Antony P.X, Mukhopadhyay H.K, Pillai R.M, Thanislass J, Srinivas V.M.V, Kumar R.S (2014) Methicillin-resistant staphylococci associated with bovine mastitis and their zoonotic importance. Vet. World.

[9] Pumipuntu N, Tunyong W, Chantratita N, Diraphat P, Pumirat P, Sookrung N, Chaicumpa W, Indrawattana N (2019). *Staphylococcus* spp associated with subclinical bovine mastitis in central and northeast Provinces of Thailand. PeerJ.

[10] Indrawattana N, Pumipuntu N, Suriyakhun N, Jangsangthong A, Kulpeanprasit S, Chantratita N, Sookrung N, Chaicumpa W, Buranasinsup S (2019). *Staphylococcus argenteus* from rabbits in Thailand. Microbiologyopen.

[11] Szabados F, Woloszyn J, Richter C, Kaase M, Gatermann S (2010). Identification of molecularly defined *Staphylococcus aureus* strains using matrix-assisted laser desorption/ionization-time of flight mass spectrometry and the biotyper 2.0 database. J. Med. Microbiol.

[12] Boggs S.R, Cazares L.H, Drake R (2012). Characterization of a *Staphylococcus aureus*USA 300 protein signature using matrix-assisted laser desorption/ionization time-of-flight mass spectrometry. J. Med. Microbiol.

[13] Lu J.J, Tsai F.J, Ho C.M, Liu Y.C, Chen C.J (2012). Peptide biomarker discovery for identification of methicillin-resistant and vancomycin-intermediate *Staphylococcus aureus* strains by MALDI-TOF. Anal. Chem.

[14] Holt D.C, Holden M.T.G, Tong S.Y.C, Castillo-Ramirez S, Clarke L, Quail M.A, Currie B.J, Parkhill J, Bentley S.D, Feil E.J, Giffard P.M (2011). A very early-branching *Staphylococcus aureus* lineage lacking the carotenoid pigment staphyloxanthin. Genome Biol. Evol.

[15] Thaipadungpanit J, Amornchai P, Nickerson E.K, Wongsuvan G, Wuthiekanun V, Limmathurotsakul D, Peacock S.J (2015). Clinical and molecular epidemiology of *Staphylococcus argenteus* infections in Thailand. J. Clin. Microbiol.

[16] Chantratita N, Wikraiphat C, Tandhavanant S, Wongsuvan G, Ariyaprasert P, Suntornsut P, Thaipadungpanit J, Teerawattanasook N, Jutrakul Y, Srisurat N, Chaimanee P, Anukunananchai J, Phiphitaporn S, Srisamang P, Chetchotisakd P, West T.E, Peacock S.J (2016). Comparison of community-onset *Staphylococcus argenteus* and *Staphylococcus aureus* sepsis in Thailand:A prospective multicentre observational study. Clin. Microbiol. Infect.

[17] Pumipuntu N, Kulpeanprasit S, Santajit S, Tunyong W, Kong-Ngoen T, Hinthong W, Indrawattana N (2017). Screening method for *Staphylococcus aureus* identification in subclinical bovine mastitis from dairy farms. Vet. World.

[18] Zschock M, Kloppert B, Wolter W, Hamann H.P, Lammler C.H (2005). Pattern of enterotoxin genes seg, seh, sei and sejpositive *Staphylococcus aureus* isolated from bovine mastitis. Vet. Microbiol.

[19] Witaya S (2011). Epidemiology of subclinical mastitis and their antibacterial susceptibility in smallholder dairy farms, Chiang Mai Province, Thailand. J. Anim. Vet. Adv.

[20] Hosseinzadeh S, Saei H.D (2014). Staphylococcal species associated with bovine mastitis in the North West of Iran:Emerging of coagulase-negative staphylococci. Int. J. Vet. Sci. Med.

[21] Persson Y, Nyman A.K, Andersson U.G (2011). Etiology and antimicrobial susceptibility of udder pathogens from cases of subclinical mastitis in dairy cows in Sweden. Acta Vet. Scand.

[22] Bakir M, Sabrina R, Toufik M (2011). Antibacterial susceptibility profiles of sub-clinical mastitis pathogens isolated from cows in Batna and Setif governorates (East of Algeria). Vet. World.

[23] Kaliwal B.B, Sadashiv S.O, Kurjogi M.M, Sanakal R.D (2011). Prevalence and antimicrobial susceptibility of coagulase-negative staphylococci isolated from bovine mastitis. Vet. World.

[24] Huang Y.L, Sun Q.L, Li J.P, Hu Y.Y, Zhou H.W, Zhang R (2019). Evaluation of an in-house MALDI-TOF MS rapid diagnostic method for direct identification of micro-organisms from blood cultures. J. Med. Microbiol.

[25] Tong S.Y, Schaumburg F, Ellington M.J, Corander J, Pichon B, Leendertz F, Bentley S.D, Parkhill J, Holt D.C, Peters G, Giffard P.M (2015). Novel staphylococcal species that form part of a *Staphylococcus aureus* related complex:The non-pigmented *Staphylococcus argenteus* sp nov. and the non-human primate-associated *Staphylococcus schweitzeri* sp. nov. Int. J. Syst. Evol. Microbiol.

